# Detection of renal dysfunction by point of care creatinine testing in patients undergoing peripheral MR angiography

**DOI:** 10.1186/1532-429X-13-S1-P173

**Published:** 2011-02-02

**Authors:** Kevin R Kalisz, Amir H Davarpanah, Asad A Usman, Jeremy D Collins, Timothy J Carroll, James C Carr

**Affiliations:** 1 Northwestern University, Chicago, IL, USA

## Introduction

To analyze the effectiveness of pre-study questionnaires in identifying at-risk patients as well as estimate the prevalence of chronic kidney disease (CKD), nephrogenic systemic fibrosis (NSF) risk factors, and other co-morbidities in patients scheduled to undergo magnetic resonance angiography (MRA) gadonlinium-based contrast agents (GBCA) studies.

## Purpose

Nephrogenic systemic fibrosis (NSF) is a rare, but serious, disease that is highly linked to the use of GBCAs in patients with renal insufficiency. Identification of these patients is considered vital prior to performing any such study. Thus, many institutions have administered pre-study questionnaires to patients in efforts to determine the degree of any pre-existing renal disease. However, identification of patients with renal dysfunction using this method has proven challenging due to many patients denying any history of renal disease on patient questionnaires, despite having low glomerular filtration rate (GFR) values. As a result, some institutions have instituted same-day point of care (POC) serum creatinine testing to estimate GFR values for all patients scheduled to undergo GBCA studies. In this study, we sought to quantify the effectiveness of these questionnaires relative to same-day POC data in identifying patients with renal insufficiency.

## Methods

This study was based upon a HIPAA-compliant and IRB-approved retrospective review of patients’ records, and informed consent of patients was not required. Patent demographics, co-morbidities, contrast type, POC serum creatinine values, and responses to pre-study questionnaires regarding knowledge of patients’ history of renal dysfunction were recorded for all patients undergoing a lower extremity magnetic resonance angiography exam in a single year. Patients were compared with respect to demographic, co-morbidity, and contrast data according to their CKD status. Patient questionnaire results were also compared to their POC-based CKD classification.

## Results

**Figure 1:**
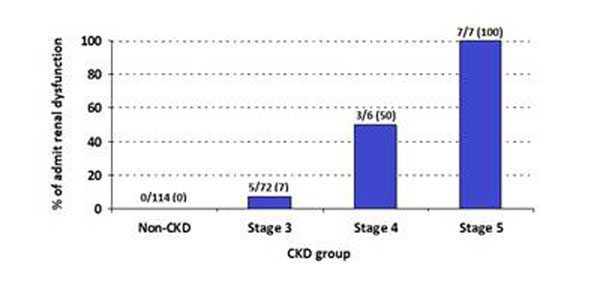
Admission of renal dysfunction by CKD group

Of 199 total patients, 72 patients had stage 3 CKD, 6 patients had stage 4 CKD, and 7 patients had stage 5 CKD. Co-morbidities including transplant status, presence of diabetes, hypertension, coronary artery disease, and smoking status showed significant differences among the CKD groups. Only 6.9% of stage 3 patients were aware of any history of renal dysfunction, whereas 50% of stage 4 and 100% of stage 5 patients admitted any history of renal dysfunction via questinonnaires (Figure 1).

## Conclusion

A pre-study questionnaire alone cannot be relied upon in screening at-risk patients, and POC creatinine testing can more reliably detect renal dysfunction in patients scheduled to undergo MRA GBCA studies.

